# Deep phenotyping of T regulatory cells in psoriatic arthritis highlights targetable mechanisms of disease

**DOI:** 10.1016/j.jbc.2024.108059

**Published:** 2024-12-09

**Authors:** Tegan McTaggart, Jing Xuan Lim, Katie J. Smith, Bronagh Heaney, David McDonald, Gillian Hulme, Rafiqul Hussain, Jonathan Coxhead, Abbie EA. Degnan, John Isaacs, Arthur Pratt, Shoba Amarnath

**Affiliations:** 1 Biosciences Institute, Newcastle University, Newcastle Upon Tyne, UK; 2 NIHR, Biomedical Research Centre, Newcastle Upon Tyne, UK; 3 Translational and Clinical Research Institute, The Medical School, Newcastle University, Newcastle Upon Tyne, UK; 4 Department of Rheumatology, Newcastle Upon Tyne Hospitals NHS Foundation Trust, Newcastle Upon Tyne, UK

**Keywords:** PD-1, PsA, Tregs, OCP, BD Rhapsody

## Abstract

Regulatory T cells (Tregs) are immune regulatory T cells that are vital for controlling inflammation. The role of Tregs in inflammatory diseases namely psoriatic arthritis (PsA) is still poorly understood. The underlying reason being a lack of robust unbiased analysis to test the immune regulatory phenotype of human Tregs. Here, we propose that checkpoint receptors can identify functional Tregs in PsA. Using unbiased BD Rhapsody single-cell analysis, we have analyzed the expression pattern of checkpoint receptors in Tregs and found that PsA Tregs are enriched in the expression of CTLA4, TIGIT, PD-1, and GITR while TIM3 was downregulated. Furthermore, PD-1^+^ Tregs in PsA had an increased type 1 phenotype and expressed the protease asparaginyl endopeptidase. By harnessing the PD-1 signaling pathway and inhibiting asparaginyl endopeptidase, PsA Treg function was significantly enhanced in *in vitro* suppressor assays. Next, we interrogated the cell interaction pathways of Tregs in PsA and found a diminished crosstalk with circulating osteoclast precursors through the CD244–CD48 coreceptor pathways. Therapeutically, PsA Treg function could be enhanced by modulating PD-1 and osteoclast interactions. Our study suggests that unconventional immune cell crosstalk with Tregs is severely diminished in PsA.

Regulatory T cells (Tregs) are a subset of CD4^+^ T cells that are critical for maintaining immune homeostasis. Human Tregs can be differentiated into multiple subtypes based on the expression of the Treg master transcription factor FOXP3, CD25 (IL-2R), CD127 (IL-7R), Helios, and CD45RA (1). Tregs also express a number of coreceptors such as CTLA4 (2), TIM-3 (3), and TIGIT (4, 5), which play a role in augmenting Treg cell function. Of these, PD-1 and CTLA4 play a significant role in inhibiting antitumor immunity (6); PD-1 can also stabilize FOXP3 function (7, 8). PD-1 receptor activation through programmed death ligand-1 ligation was shown to stabilize FOXP3 in human T cells that also expressed the transcription factor, Tbet, a master regulator of T helper type 1 (Th1) differentiation (9). We have previously found that PD-1 expression was increased in murine Tbet^+^Tregs (10) and drove Treg cell functional plasticity through a protease called asparaginyl endopeptidase (AEP) (8). On activating PD-1 signaling through PD-L1, AEP is inhibited, maintaining FOXP3 protein expression in both mice and human Tregs (9, 10). A similar phenotype has also been demonstrated in HIV Tregs (11). Taken together, there may be a population of Tregs in autoimmune conditions, whose function may be therapeutically harnessed through activation of the PD-1 signaling pathway.

Psoriatic arthritis (PsA) is an inflammatory joint disease characterized by joint destruction and bone loss. T cells play an important role in the pathogenesis of this disease, with T helper type 17 (Th17) cells being a crucial population in driving disease phenotype (12, 13). Th17 cells are characterized by the expression of the transcription factor RORC and cytokine IL-17. Like Tregs, a subset can coexpress Tbet, which is linked with IL-12/23 stimulation and associated with inflammatory disease (14). Evidence for the link between PsA and Tbet^+^ Th17 cells is provided by the efficacy of both IL-17 and IL-12/IL-23R targeting drugs secukinumab, bimekizumab, and ustekinumab in treating PsA (15, 16). These data suggest a Tbet-mediated inflammatory process within PsA, which is yet to be fully deciphered and like Treg cells, may be regulated by the PD-1 pathway.

The role of Tregs in the pathophysiology of PsA is controversial. For example, some studies indicate that Treg-mediated enhancement of Th17 migration in this disease may be reversed using antitumor necrosis factor (TNF) antibodies (17). In other studies, Tregs have been implicated to acquire an IL-17 phenotype with the expression of CD161 marker (18). In addition, studies implicate coreceptor expression on Tregs to PsA disease with ICOS expression on Tregs correlating positively with PsA disease score (18). A recent article on Tregs in PsA has shown that peripheral blood and joints consist of five different Treg subsets with the CD161^+^ Tregs being enriched for LAG3 and IL-10 along with PD-1 expression. The LAG3 expression on CD161^+^ Tregs directly inhibits IL-12–IL-23 and TNF secretion by patient monocytes in spondyloarthritis, and this population was conserved in PsA (19). These studies contradict the hypothesis that CD161^+^ Tregs are inflammatory in phenotype (20, 21) and therefore warrants a thorough understanding of how coreceptors drive Treg function in inflammatory disease. Indeed, all five different subsets identified in the study expressed PD-1, CTLA4, TIGIT, and GITR (19) to some extent, suggesting that these coreceptors could play a role in Treg function in PsA. However, the crosstalk of Tregs with other immune cells in PsA remains largely unexplored. This key component of Treg biology in PsA therefore requires further investigation.

In this study, by performing unbiased analysis of peripheral blood mononuclear cells (PBMCs), we have investigated several aspects of Treg biology, namely (1) coreceptor expression patterns in PsA Tregs, (2) functional subtypes of Tregs, and (3) Treg crosstalk with osteoclast precursors (OCPs). We found that PsA Tregs consisted of a subset that expressed PD-1, which also possessed a type 1 phenotype. Harnessing PD-1 molecular signaling enhanced PsA Treg function in *in vitro* suppressor assays. Next, we found using unbiased cell communication analysis, that PsA Tregs showed diminished communication with CD16^+^ OCP. Several communication pathways with OCP were interrupted in PsA Tregs compared with healthy Tregs and may contribute toward disease. Enhancing this communication may provide novel therapies in the treatment of PsA.

## Results

### Single-cell BD Rhapsody profiling of PBMCs from PsA shows heterogeneous conventional coreceptor expression in Tregs

**Figure 1:**
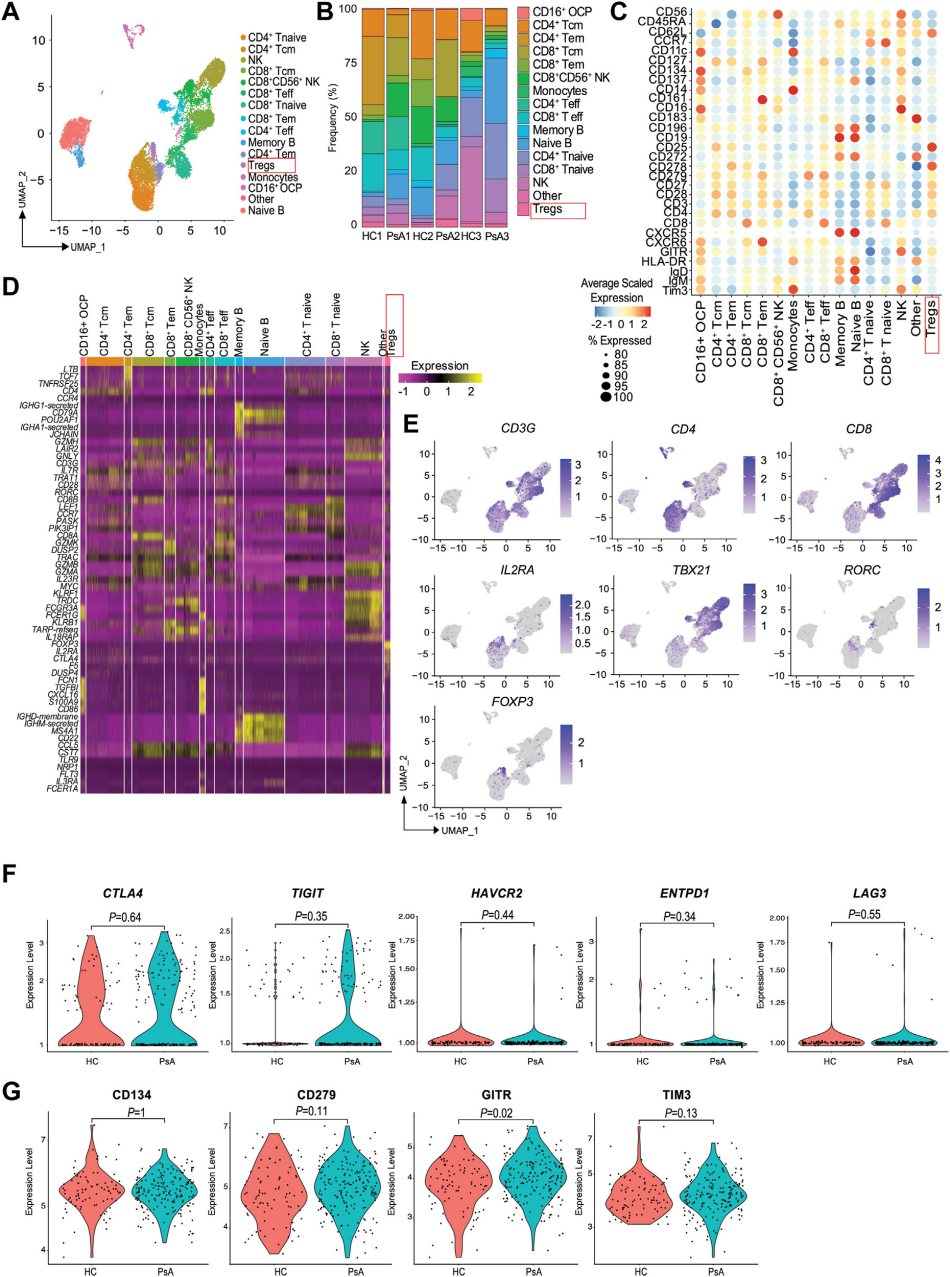
**Single-cell analysis of peripheral blood immune cells in psoriatic arthritis (PsA).** PBMCs from healthy controls (HCs) and PsA were subjected to single-cell analysis using 30 AbSeq antibodies and a 399 immune gene panel. Immune cell populations within PBMCs were identified using UMAP analysis (*A*), and the frequency of each population within each sample was measured (*B*). AbSeq antibodies were used for defining the various immune cell populations (*C*), and then unbiased clustering was done by transcript analysis using a heatmap, with the top five genes shown per cluster (*D*). UMAPs of gene expression defining T cells and Tregs (*E*) are shown. Treg canonical coinhibitory receptor gene expression values (*F*) and protein expression values of PD-1 (CD279), GITR, TIM3, and CD134 (*G*) on Tregs are shown as violin plots. Data shown are from n = 3 samples for HC and PsA, 6038 and 8253 cells analyzed for HC and PsA, respectively. Statistical analysis for violin plots were done using the Wilcoxon test. The expression legend in *C* and *D* indicates the average scaled gene expression of the AbSeq protein or mRNA transcript for each cell type, with *red* indicating the highest expression and *blue* indicating the lowest expression. *C*, the size of the *circle* refers to the number of cells within an immune cell population that express a given AbSeq. PBMC, peripheral blood mononuclear cell; Treg, regulatory T cell; UMAP, uniform manifold approximation and projection.

In order to characterize the coreceptor protein and transcriptional landscape of immune cells in PsA, we performed a BD Rhapsody analysis, as previously published by us (22), on CD45^+^ sorted PBMCs from age-matched healthy controls (HCs) and PsA patients. We first identified immune cell populations, visualized by uniform manifold approximation and projection analysis (Fig. 1*A* and Table S1), and the frequency of cells within each cluster showed a distribution that is characteristic of human immune cell diversity (Fig. 1*B*), with no significant difference between groups. Using AbSeq protein expression, we used 30 antibodies to define immune cell populations and the expression of chemokine receptors and coreceptor expression in various cell subsets (Fig. 1*C*). Next, using transcript expression, we performed an unbiased clustering analysis and found that the heatmap correlated with AbSeq-based clustering. For example, the Treg cluster was identifiable as noted by *FOXP3* gene expression (Fig. 1, *D* and *E* and Table S2). No difference in FoxP3 protein frequency was noted between HC and PsA by flow cytometry (Fig. S1, *A* and *B*). To further confirm whether a difference in FoxP3 exon 2 existed in PsA Tregs (23, 24, 25, 26), we utilized flow cytometry analysis. No change in frequency or mean fluorescence intensity of exon 2 expression between HC and PsA was noted (Fig. S1, *C*–*E*). Next, we measured the cytokines transforming growth factor-β and IL-10 in PsA Tregs. We found no difference in the expression of any of these markers in PsA Tregs as compared with HCs (Fig. S1, *F* and *G*).

We then interrogated canonical coinhibitory receptor expression in the scRNA-Seq data. In keeping with published data, we found a significant increase in GITR (Ab) in PsA Tregs compared with HCs. Expression of PD-1 (CD279 Ab), *TIGIT*, and *CTLA4*, transcripts were enriched in PsA, but this did not reach statistical significance when compared to HC Tregs. No change in CD134, *LAG3*, *HAVCR2*, and *ENTPD1* expression was noted in PsA Tregs (Fig. 1, *F* and *G*). A decrease in TIM3 expression was noted in PsA Tregs, but again this did not reach statistical significance. Expression in each individual donor is shown in Fig. S2, *A* and *B*. These data demonstrate that Tregs in PsA express canonical coinhibitory receptors of potential relevance for targeting disease management.

### Differential gene expression analysis identifies galectin 1 along with an increase in apoptotic pathways and cytotoxic phenotype within PsA Tregs

**Figure 2:**
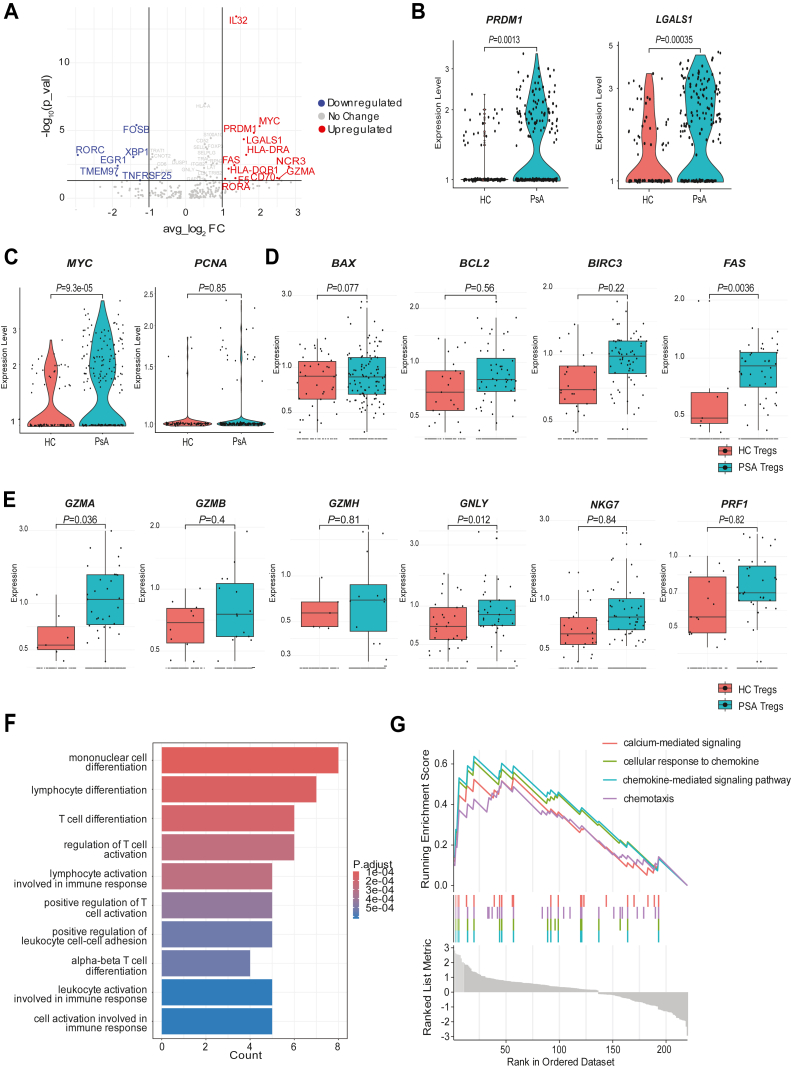
**Characterization of PsA Tregs unravels an enhanced cytotoxic phenotype and apoptotic pathways.** Volcano analysis of differential gene expression between healthy control (HC) and PsA (*A*) with upregulated and downregulated genes defined by log_2_ fold change >1 or <−1 and a *p* value <0.05. Violin plots showing statistical differences in *PRDM1* and *LGALS1* gene expression in PsA Tregs (*B*). The proliferative capacity of Tregs was measured using *MYC* and *PCNA* gene expression (*C*), Treg apoptotic phenotype was measured using canonical genes (*D*), and Treg cytotoxic phenotype is shown (*E*). Gene Ontology over-representation pathway analysis in PsA Tregs is shown (*F*), and gene set enrichment analysis shows increased activation of PsA Tregs (*G*). Statistical analysis for violin plots and box plots was done using the Wilcoxon test. PsA, psoriatic arthritis; Treg, regulatory T cell.

To identify key genes that are enriched in PsA Tregs, we performed an unbiased differential gene expression analysis visualized using a volcano plot comparing HC and PsA (Fig. 2*A* and Table S3). The data showed that PsA Tregs were significantly enriched in cytotoxic markers (*GZMA*, *FAS*), had an increase in the regulatory protein galectin-1 (*LGALS1*) along with an increase in *PRDM1* (BLIMP-1), a master regulator of IL-10 production in Tregs (Figs. 2*B* and S2*C*). However, no change in IL-10 protein was noted in PsA Tregs (Fig. S1*G*). We next analyzed the proliferative genes *MYC* and *PCNA*, finding a significant increase in *MYC* but not in *PCNA* (Fig. 2*C*). We then used Ki67 as a protein marker of cycling Tregs and measured the ability of Tregs to cycle in HC and PsA Tregs. Compared with mRNA data, a significant increase in Ki67^+^ Tregs was noted in PsA (Fig. S3, *A* and *B*), suggesting an increase in Treg cycling within PsA patients. Next, we stimulated PBMCs with αCD3 to determine the potential of Tregs to expand in PsA in response to polyclonal T-cell receptor (TCR) stimulation. No significant difference in Cell Trace Violet (CTV) dilution as a measure of proliferation was noted between HC and PsA (Fig. S3, *C* and *D*). We next analyzed the apoptotic markers in Tregs and did not find key changes in most genes, but found a significant increase in *FAS*, suggesting a regulatory function of PsA Tregs that may be driven by FAS/FASL (Fig. 2*D*). Finally, in keeping with previous reports (19), and in order to confirm the cytotoxic phenotype of PsA Tregs, we performed a violin plot analysis of a whole array of cytotoxicity genes and found PsA Tregs were enriched in cytotoxicity markers such as *GZMA* and *GNLY* (Fig. 2*E*). Taken together, these data suggest that PsA Tregs are poised for effector function, upregulating migratory properties, activation pathways along with regulatory gene networks that have the potential to inhibit other immune cells through cell–cell interaction (Galectin-1), apoptotic pathways (FASL), and cytotoxicity (*GZMA*). Across CD4^+^FoxP3^-^ T-cell clusters, we observed an upregulation of *MYC* in PsA in most subsets as well as markers of activation/memory (*SELL*, *CCR7*, and *CD69*), apoptosis (*BIRC3*), and Th1 differentiation (*TBX21*) (Fig. S4, *A*–*D*). Cytotoxic gene signatures including *GNLY*, *GZMH*, and *GZMB* were upregulated in CD8^+^ T cells in PsA compared with HCs (Fig. S4, *E*–*I*), demonstrating an enhanced CD8^+^ T-cell function in PsA in line with recent reports (27). Next, Treg pathway analysis showed an increase in the lymphocyte activation pathways in PsA Tregs with gene set enrichment analysis showing that PsA Tregs were enriched in calcium-mediated, chemokine, and chemotaxis signaling (Fig. 2, *F* and *G*, Tables S4, and S5).

### PsA Tregs possess significant type 1 phenotype

**Figure 3:**
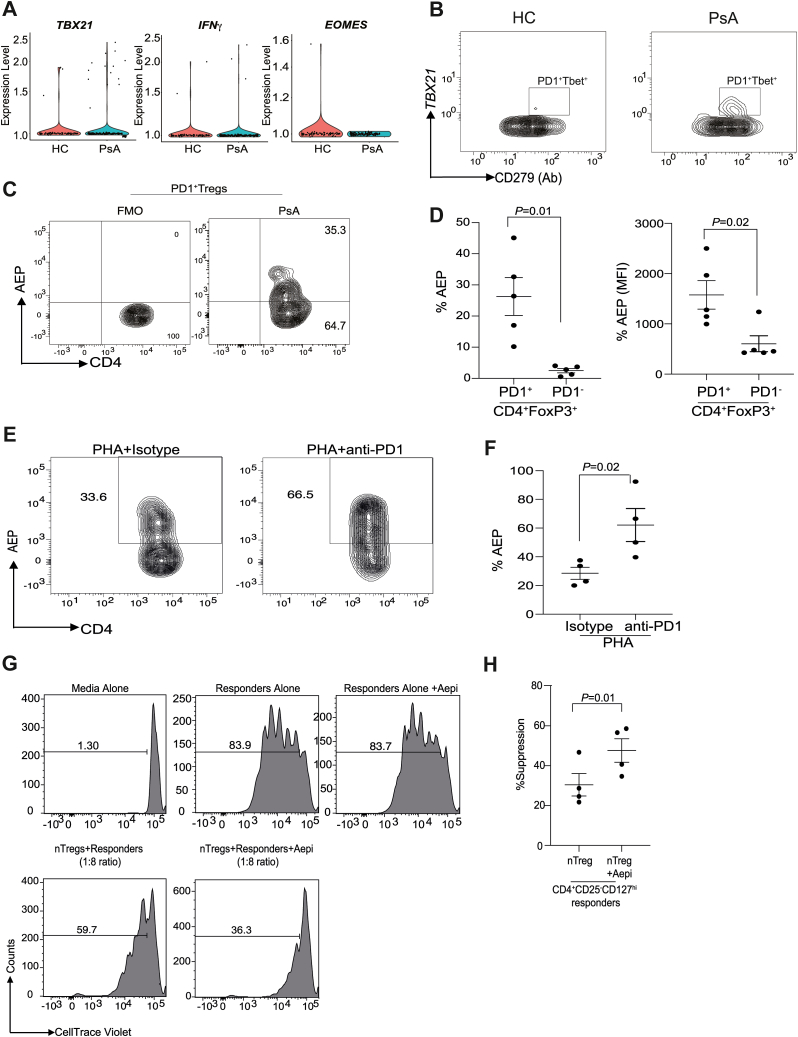
**PD-1 can be harnessed to enhance PsA Treg function.** The gene expression values of type 1 functional markers within the single-cell dataset were analyzed using violin plots (*A*). PD-1 AbSeq protein expression is shown in healthy control (HC) and PsA Tbet^+^Tregs using SeqGeq software (*B*). Human PBMCs were isolated from PsA patients and stained for CD4, FoxP3, PD-1, and active AEP. AEP frequency and protein expression was measured (*C* and *D*). Tregs from PsA were cultured for 3 days in the presence of PHA or PHA plus anti-PD-1 (*E*). An increase in AEP on PD-1 signaling blockade is shown in PsA Tregs (*F*). PsA donors were used for both responders and TReg cells. CD4^+^CD25^-^CD127^hi^ cells were labeled with CellTrace Violet and expanded in the presence of Treg suppression inspector for 5 days either alone or in the presence of Tregs, Tregs plus AEP inhibitor. At day 5, proliferation was measured by flow cytometry. Representative flow plot is shown (*G*) and summary of Treg suppression in the absence and presence of AEP inhibitor (*H*), n = 4 donors. Data shown are mean + SEM and a paired Student's *t* test was performed to determine statistical significance. Statistical analysis for violin plots were done using the Wilcoxon test. AEP, asparaginyl endopeptidase; PBMC, peripheral blood mononuclear cell; PHA, phytohemagglutinin; PsA, psoriatic arthritis; Treg, regulatory T cell.

Given the success of ustekinumab in PsA and its role in blocking IL-12–IL-23R signaling, there remains an unknown role for Tbet in PsA pathogenesis. Hence, we interrogated the Th1 phenotype of PsA Tregs by testing if this population was regulated by the PD-1 signaling pathway. We found that PsA Tregs expressed *TBX21* (Tbet) and *IFNG* with minimal expression of *EOMES* (Fig. 3*A*). Tbet expression was solely restricted to PD-1^+^ PsA Tregs (Fig. 3*B*). Given that PD-1 (CD279) protein expression was also noted in PsA Tregs (Fig. 1*G*), we measured if active AEP protease was expressed in PD-1^+^ Tregs in PsA. We first confirmed that Tbet^+^ Tregs expressed PD-1 and found that all the Tbet-expressing cells coexpressed PD-1 in PsA Tregs. By contrast, this population was absent in HCs (Fig. 3*B*). We found that PD-1^+^ Tregs in PsA expressed active AEP (Fig. 3, *C* and *D*), and blocking the PD-1 pathway in Tregs significantly enhanced AEP expression (Fig. 3, *E* and *F*). We next tested if we could enhance PsA Treg function by blocking AEP and found that blocking AEP enhanced PsA Treg function using an *in vitro* suppression assay (Fig. 3, *G* and *H*). Taken together, we propose that PsA Tregs have enhanced Tbet expression (Fig. 3*A*), suggesting the existence of functional plasticity, which can drive their dysfunction. Harnessing PD-1 can overcome this functional defect in PsA Tregs.

### Pseudotime analysis between HCs and PsA highlights altered Treg differentiation programs

**Figure 4:**
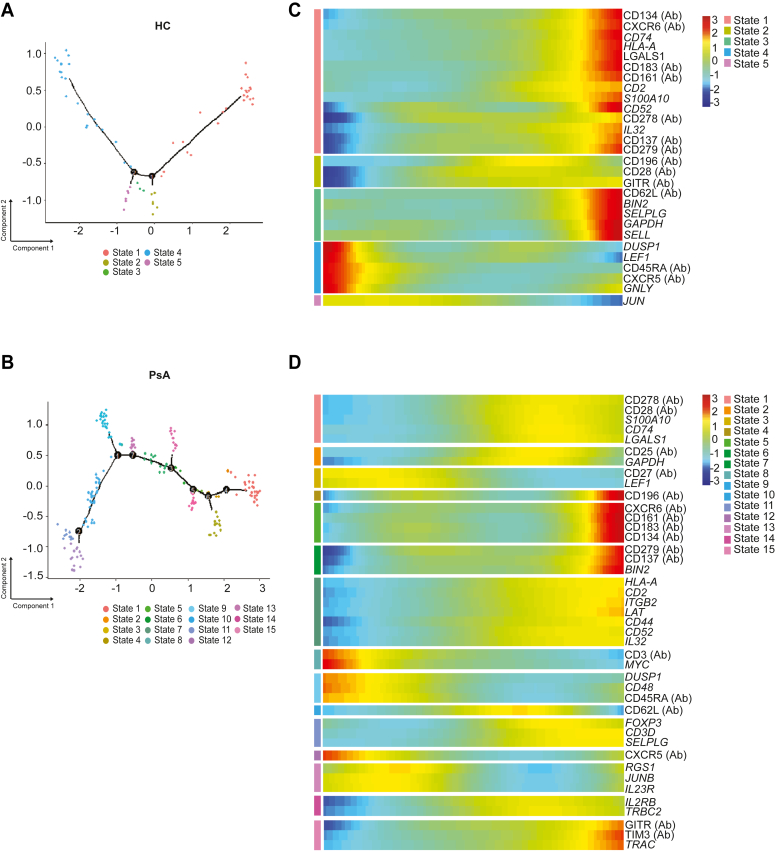
**Monocle and pseudotime analysis demonstrate an upregulation of Treg differentiation states in PsA.** The single-cell data were analyzed using the monocle plugin on SeqGeq software to predict differentiation states of Tregs across pseudotime. For monocle analysis, we excluded non-CD4^+^ T-cell AbSeq. In healthy control (HC) Tregs, two nodes of differentiation correspond to five differentiation states (*A*), shown as branched trajectory graphs. In PsA Tregs, seven points of differentiation and 15 states (*B*) are shown. Nodes of differentiation are represented as *numbered black dots* and are points where cells determine their fate progressing onto one branch or the other. Pseudotemporal heatmap visualization of differentially expressed genes for each state in HC (*C*) and PsA (*D*) Tregs. Each row is a particular gene, specific to a defined state. PsA, psoriatic arthritis; Treg, regulatory T cell.

We next determined the differentiation programs of Tregs in PsA using monocle and pseudotime trajectory analysis (28). Using the SeqGeq software monocle plugin, we found that HC Tregs had two different nodes of differentiation points (Fig. 4*A*), whereas PsA Tregs had seven points of divergence (Fig. 4*B*). Pseudotime quantitative analysis demonstrated that in HCs several Treg differentiation states were apparent, each defined by a unique gene expression profile that could contribute toward Treg function (Fig. 4, *A* and *C* and Table S5). In PsA, we found a substantial increase in differentiation states of Tregs, highlighting the existence of several functional subsets of peripheral Tregs within this population (Five states in HC *versus* 15 states in PsA) (Fig. 4, *B* and *D* and Table S6). Each state in PsA Tregs was enriched for gene sets that were different to HCs (Fig. S5), hence suggesting that pseudotime analysis of PsA Tregs highlights the various differentiation programs that can be activated during inflammation. It is worth noting that the monocle analysis is an unbiased predictive analysis that can suggest the existence of various Treg states within PsA, but further confirmation of these states is warranted. Nevertheless, identifying such heterogeneity is key to understanding the role of Tregs in disease.

### Predictive CellChat analysis on PsA Tregs shows multiple unique communication patterns compared with HCs

**Figure 5:**
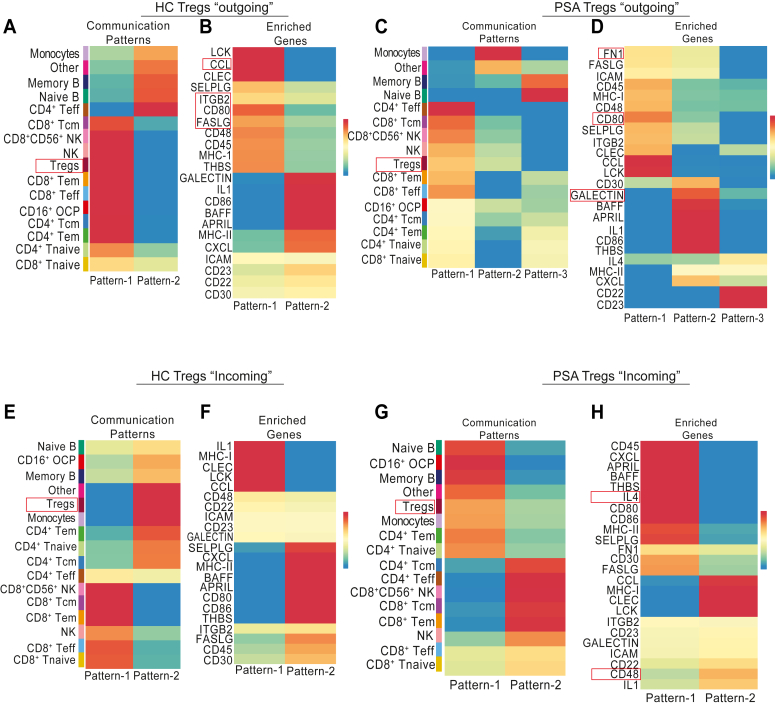
**CellChat analysis demonstrates an increase in FN1-mediated cell network crosstalk patterns in Tregs.** CellChat analysis was carried out using R studio on the single-cell dataset to predict the patterns of outgoing (sending) and incoming (receiving) signals across all immune cell populations. The outgoing cell communication patterns (*A*) and specific signaling pathways driving these communication patterns in healthy controls (HC) (*B*) are shown. Similarly, the outgoing communication pattern (*C*) and enriched signaling pathways in PsA (*D*). Incoming signaling patterns (*E*) and signaling pathways (*F*) in HCs and PsA (*G* and *H*) are plotted as heatmaps. *Red* indicates a higher contribution of a cell cluster or specific signaling pathway to each pattern. FN1, fibronectin 1; PsA, psoriatic arthritis; Treg, regulatory T cell.

Using the previously defined clusters of immune cells, we next analyzed predicted cell interactions between each cell cluster through expression of ligand-receptor pairs identified by “CellChat” analysis (29). This identified two distinct patterns of outgoing cell interactions in HC PBMCs. Pattern 1 was seen in most subtypes of CD8^+^ T cells and CD4^+^ T cells, including Tregs, but excluding CD4^+^ T effector cells that were associated with pattern 2 cell ligand–receptor interactions (Fig. 5*A*). Enriched genes that contribute to pattern 1 and pattern 2 for HC PBMCs are listed in Figure 5*B*. Pattern 1 genes associated with Treg cells included members of the FAS/FASL pathway, CTLA4/CD80, and CCL migratory signals (Fig. 5*B*) in line with established patterns of normal Treg communication with adjacent immune cells. When we performed similar analysis in immune cells from PsA patients, we identified three distinct patterns. PsA patient Tregs possessed two different outgoing communication patterns (Fig. 5*C*), one enriched in CTLA4/CD80 and the other in GALECTIN (Fig. 5*D*), identifying two differentiation programs in line with our pseudotime analysis. Of significant note is the upregulation of the fibronectin 1 (*FN1*)-mediated outgoing communication pattern seen in patterns 1 and 2 in cells from PsA patients. Fibronectins are an essential part of the extracellular matrix and play an important role in inflammation in osteoarthritis (30). In psoriasis, FN1 was highly expressed in synovial biopsies suggesting its role in the pathogenesis of the disease (31). Here, our *in silico* unbiased analysis suggests that *FN1* may be involved in driving immune system function in PsA being one of the novel signaling pathways that is unique to PsA. Next, we analyzed the signals that may activate Tregs by other immune cells and termed “incoming signals”. Here, we found that HC Tregs were associated with a single pattern (pattern 2) and PsA Tregs, had dual patterns of incoming signals (pattern 1 and to a lesser extent pattern 2) (Fig. 5, *E*–*H*). Of note, in addition to *FN1* and IL-4, CD48 signaling was enhanced in PsA patient Tregs (Fig. 5*H*). It should be noted that these are predictive pattern analyses, and further confirmation using ligand pair analysis is needed to determine specific ligands and receptors that can interact with these pathways. Confirmation of protein expression followed by functional assays is required.

### Predictive CellChat ligand-pair analysis on PsA Tregs suggests a failure of Tregs to communicate with OCP through the CD48–CD244 axis, suggesting that the key dysfunction of PsA Tregs is associated with bone loss

**Figure 6:**
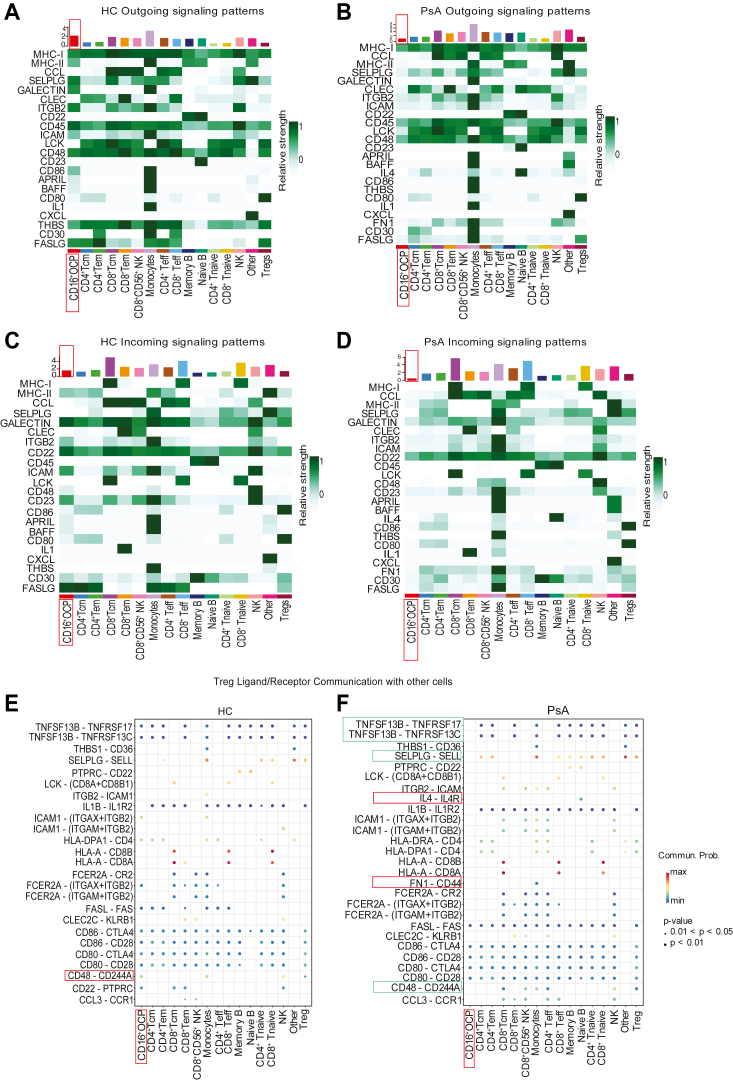
**CellChat analysis identifies specific ligand–receptor pairs that prevent CD16**^**+**^**OCP and Treg crosstalk in PsA.** On identifying the patterns of communication, further analysis was performed to specifically identify the contribution of each immune cell and the respective gene pathways involved in sending (outgoing) and receiving (incoming) signals in healthy control (HC) and PsA. Heatmap visualizing the outgoing signaling contribution of each cell type in HC (*A*) and PsA (*B*). Similarly, the incoming signals for all immune cell clusters in both HC (*C*) and PsA (*D*) are shown. *Color* reflects strength of signal; *top bar* represents cell contribution to overall communications. Next, in-depth specific gene set analysis was performed to predict the contribution of the ligand–receptor pairs that were disrupted in Treg crosstalk with other cells in HC and PsA (*E* and *F*). *Dot color* represents communication probability, and *dot size* reflects *p* value. PsA, psoriatic arthritis; Treg, regulatory T cell.

We next analyzed the predicted strength of the communication between the immune cells in healthy and PsA patient PBMCs using cell–cell interaction gene heatmaps. Here, we found that in contrast to HCs, CD16^+^ OCPs from PsA patients failed to contribute toward outgoing (Fig. 6, *A* and *B*) and incoming signals (Fig. 6, *C* and *D*), thereby suggesting diminished crosstalk with neighboring cells. We found that HC human Tregs possessed ligand–receptor expression that suggested a robust communication with all the subsets identified in the single-cell analysis (Fig. 6*E*). In contrast, PsA Treg analysis suggested a diminished crosstalk with CD16^+^ OCPs (Fig. 6*F*) along with an increased communication with B cells and monocytes in PsA through IL4–IL4R and FN1–CD44 axis.

Focusing on diminished communication pathways as a measure of Treg dysfunction, we further explored the ligand–receptor pair interaction between OCP and Tregs. It has been shown that CD16^+^CD14^+^ monocytes/macrophages are a source of OCPs in PsA, and this population is key in bone resorption, a major cause of morbidity in psoriatic disease (32, 33, 34). Our data suggest that one major dysregulation within Tregs in PsA could be associated with OCP, whereby Tregs may fail to control this population. We next tested if the lack of communication was singularly affected by the CTLA4/CD80–CD86 pathway and found this not to be the case (Fig. 6*F*), which was in contrast with the literature that abatacept can prevent osteoclastogenesis (35, 36). In contrast to CTLA4 signaling, we found changes in several other communication networks in PsA Tregs when compared with healthy Tregs, such as TNFR, CD48, and SELPLG. Hence, we have a Treg signature that could be used to determine unchecked OCP regulation in PsA patients. Hence, a key aspect of Treg dysregulation in PsA could be through OCP. It has been shown using *in vitro* functional experiments that Tregs are capable of suppressing OCP (37) and can protect from local and systemic bone destruction in arthritis (38). Here, our predictive analysis suggests that the main contribution of Tregs to PsA is through the OCP axis. In order to support our unbiased CellChat analysis, we then focused on the CD48–CD244 axis dysregulation. We first quantitatively analyzed the mRNA transcripts of CD48 on OCPs. We found significant downregulation of CD48 mRNA in OCPs in PsA (Fig. S6*A*). Flow cytometry analysis was performed to measure protein expression, and similar to mRNA, a significant decrease in CD48 protein was noted in OCP (Fig. S6, *A* and *B*). No difference in CD244, the ligand for CD48 was found in Tregs (Fig. S6, *C* and *D*). Hence, by downregulating CD48, a key OCP/Treg-mediated regulation could be subverted in PsA. It should be noted that functional assays are required to further confirm this crosstalk. Therefore, using our dataset, emerging new patterns of Treg phenotype and function in PsA can be discerned.

## Discussion

Tregs are vital for protecting the host against unwarranted inflammation, thereby maintaining immune homeostasis. In inflammatory diseases, Treg function is important for resolving underlying inflammation and has been extensively studied. The role of Treg dysfunction and plasticity is not clearly defined in psoriatic disease (39). Although phenotypic characterization studies of Tregs in PsA have been carried out (17, 18), their contribution in PsA disease remains incompletely understood. Here, using an unbiased single-cell approach, we provide evidence that Treg dysfunction in PsA is attributed to functional plasticity and dysregulated crosstalk with monocytes, B cells, and OCPs. Our work comprehensively defines the coinhibitory receptor, cytokine, chemokine, and receptor–ligand interaction landscape of PsA Tregs in the blood using both protein and transcript expression. To our knowledge, this is the first report to perform unbiased analysis on PsA immune cells and report this missing link between immunity and bone remodeling. Unlike tissue-resident CD8 and CD4 T-cell subsets, Tregs do not acquire a tissue-residency phenotype (40), hence our work on circulating Tregs can functionally define the immune-suppressive phenotype of Tregs in PsA.

We found that, like previous work, PsA Tregs have a similar expression pattern of conventional coinhibitory receptors, namely CTLA4, TIGIT (19), and PD-1 protein expression. LAG3 expression was only noted in a small fraction of cells, whereas TIM3 expression was decreased in PsA patient Tregs. Hence, using unbiased analysis, we report that PsA Tregs are enriched in GITR, PD-1, and GALECTIN-1 coreceptor signals. Of note, PsA PD-1^+^Tregs in the blood also exhibited enhanced *TBX21* expression, suggesting a functional plasticity program that is driven by PD-1. Indeed, a similar correlative phenotype has been found to be associated with ICOS in Tregs within arthritic syndromes (18, 41, 42, 43). We conclude that coinhibitory receptors can mark dysfunctional Tregs in autoimmunity, and targeting these pathways can be beneficial in enhancing Treg function. We have previously shown that PD-1^+^Tbet^+^Tregs upregulated the AEP protease, which in turn can degrade both mouse and human FoxP3 protein. Ligating PD-1 with programed death ligand-1 can prevent the functional plasticity of Tbet^+^Tregs into Th1 cells (8). Here, we further demonstrate that this mechanism is active in PsA whereby blocking AEP enhanced PsA Treg suppressive function, and this pathway was controlled by PD-1. Hence, PD-1 could be harnessed for enhancing Treg function in PsA. To our knowledge, this is the first study to show that PsA Tregs express a unique set of coinhibitory receptors, and these can be harnessed for enhancing Treg function.

In addition to functional plasticity, our data also show that the differentiation program of PsA Tregs does not follow the same pattern as HCs. The monocle and pseudotime analysis suggest that there are several nodes of differentiation present in PsA Tregs as compared with healthy Tregs, and this could result in 15 different Treg states as compared with five in normal HCs. Hence, this predictive analysis may suggest that a differential phenotype exists in PsA Tregs, which could drive their ability to inhibit various effector T cells. To date, there is no single-cell dataset on PsA Tregs or PsA immune cells that has evaluated differentiation programming of T cells in an unbiased manner (19, 27, 44). The current resource addresses the knowledge gap that currently exists in understanding immune cell differentiation in PsA pathogenesis.

The receptor–ligand pair analysis confirmed the monocle dataset, whereby PsA Tregs possessed several patterns of outgoing signaling pathways as compared to HCs, and the key pathway upregulated in PsA Tregs was driven by FN1, CD48, and IL-4. We found using receptor–ligand pair analysis that FN1 communication occurred with monocytes, suggesting that PsA immune cells may communicate *via* FN1 signals with monocytes and IL4–IL4R signals with B cells.

Of interest to our study on Treg diminished communication networks, we found that there was significant paucity in signals that arose from OCPs in PsA, and Treg crosstalk with OCP was significantly reduced. Taken together, we propose that PsA Tregs are important in controlling unconventional tissue destroying precursor cells in autoimmunity, and this crosstalk is fundamentally disrupted. Both OCP (32, 33) and FN1 (30) are implicated in arthritic disease, and here, we show that this crosstalk may be controlled by Tregs to some extent. It should be noted that our unbiased predictive analysis requires further confirmation, nevertheless it identifies previously unknown communication patterns in PsA Tregs. It also confirms that mRNA data correlates with protein expression of CD48 in OCPs, thereby suggesting that the CellChat analysis can reveal protein–protein interactions that were previously unexplored.

A major caveat of our study is the lack of TCR clone information on Tregs in PsA. Given the extensive single-cell datasets on T cells in PsA (27, 44) it is clear that T-cell clonotypic information is valuable to understand the inflammatory trajectory in PsA. However, lack of TCR information does not affect our study conclusion, whereby we observed that the key dysregulation of PsA Tregs may lie in their functional plasticity and their crosstalk with OCPs.

Furthermore, our dataset demonstrates that it is important to understand the crosstalk of Tregs with unconventional immune cells, tissue disruptive, and tissue protective precursors in autoimmunity. The major drawback in defining the role of Tregs in autoimmunity arises from this lack of knowledge, since most reports are T-cell centric, thereby not resolving the tissue protective role of Tregs. It is well established that Tregs are key for tissue repair (45), and perhaps in autoimmune conditions such as PsA, this function of Tregs is disrupted. A similar phenotype of Tregs could exist in other arthritic diseases that warrants further investigation.

In summary, our study defines several regulatory pathways in Tregs that can be harnessed through modulation of coreceptors and highlights the translational potential of coinhibitory receptors in autoimmune cell therapy.

## Experimental procedures

### Patient samples

Patients with recent onset PsA were enrolled from the Northeast Early Arthritis Cohort, a single center inception cohort of patients referred from primary care with suspected inflammatory arthritis. PsA diagnosis was confirmed by a consultant rheumatologist with reference to current classification criteria (46), and blood was drawn prior to commencement of immunomodulatory therapies, including corticosteroids. All subjects provided written informed consent for inclusion in the study; ethical approval was obtained from the Newcastle and North Tyneside 2 research ethics committee, UK (12/NE/0251). PsA patients recruited had a median age range of 50.5 (20–77), with 66% of the donors being female. The median tender joint count of 5 (0–52), swollen joint count of 2 (0–21), and an erythrocyte sedimentation rate of 14 (2–90) and C-reactive protein of 6.5 (1–29). PsA patients recruited for BD Rhapsody scRNA-Seq, had a median age range of 34 (24–48), with two of three donors being female and one with a positive RF status. The median erythrocyte sedimentation rate was 22 (22–42), and the C-reactive protein was 5 (5–19). HCs were obtained from the blood bank as leukapheresis products or bled within Newcastle University under normal control ethics (12/NE/0121). All human studies conducted abide by the Declaration of Helsinki principles.

### PBMC isolation

PBMCs were isolated from both the leukapheresis products, normal blood and blood from PsA patients as follows. The leukapheresis product was flushed out of the cone using sterile 1× PBS and then washed twice with 1× PBS. Then, leukapheresis product or blood from PsA and normal donors were diluted at a 1:1 ratio with sterile PBS and layered on top of the lymphocyte separation medium followed by centrifugation for 30 min at 600*g* with brake off. The PBMC interphase was then collected, washed twice with 1× PBS, and then used for downstream analysis.

### Cell staining and sorting

Single-cell RNA sequencing was performed using the BD Rhapsody single-cell Analysis system (BD Biosciences) according to the manufacturer’s protocol. Patient samples were thawed and stained with phycoerythrin (PE)-conjugated antihuman CD45 (BioLegend; clone; HI30) for 30 min at 4 °C. Each sample was incubated with a unique sample tag using BD Human Single-cell Multiplexing Kit (BD Biosciences) for 20 min. Cells were then washed in PBS supplemented with fetal bovine serum and stained with 4',6-diamidino-2-phenylindole. CD45+, 4',6-diamidino-2-phenylindole- cells were sorted using a BD FACSAria Fusion Cell Sorter. After sorting, cells were pooled and incubated with TruStain FcX (BioLegend) and BD AbSeq Immune Discovery Panel antibodies (BD Biosciences; CD3, CD4, CD8, CD11 C, CD14, CD16, CD19, CD25, CD27, CD28, CD45RA, CD56, CD62L, CD127, CD134, CD137, CD161, CD183, CD185, CD186, CD196, CCR7, CD272, CD278, CD279, GITR, TIM3, HLA-DR, IgD, IgM). The cell suspension was loaded onto primed BD Rhapsody cartridges and incubated for 20 min at room temperature. Then cell capture beads were washed and loaded onto the cartridge. Following cell capture bead incubation, the cartridge was washed twice, and the cells were lysed. The cell capture beads were then retrieved, and reverse transcription was performed. The bead suspension was treated with exonuclease I and subsequently stored at 4 °C for ≤3 months.

### Single-cell library preparation and sequencing

Single-cell library preparation was performed according to the manufacturer's protocol provided by BD Biosciences. Briefly, pooled sample tag/AbSeq/mRNA library was sequenced with a read length minimum of 51bp R1 and 71bp R2, and a 200-cycle sequencing protocol was used. Pooled sequencing libraries were then sequenced on NovaSeq 6000 (Illumina) using a S2 Reagent Kit v1.5 (200-cycle).

### Preprocessing and dimensionality reduction

ScRNA-Seq FastQ files were processed using the BD Rhapsody analysis pipeline (https://www.sevenbridges.com/bdgenomics/), which generated unique molecular identifier counts. In R software (version 3.3), the Seurat package (version 5.0.3) was used to further analyze transcriptomic and protein expression data. Cells were filtered for 1200 to 60,000 unique molecular identifiers, and 55 to 200 genes were detected per cell. Multiplets and undetermined cells were removed from downstream analysis. Post quality control, we determined that the percent of Tregs in all our samples were as follows: HC-1 = 2.2%; HC2 = 1.1%; HC3 = 1.6%; PsA-1 = 1.1%; PsA-2 = 3.5%; and PsA-3 = 1.4%. This frequency correlated to the following total Treg numbers: 88 Tregs for HC (11, 24, 53 Tregs) and a total of 188 (17, 123, 48 Tregs) cells for PsA. Expression matrices were log normalized, and the top 2000 variable features were identified. Samples were integrated using an anchor set and scaled by z-score conversion. Dimensionality reduction (npcs = 50) was performed using principal component analysis, and the top 20 principal components were used to calculate uniform manifold approximation and projection. A K-nearest neighbor graph was constructed with *FindNeighbors* function (k.param = 20, dims = 1:10, annoy.metric = ‘euclidean’), and Louvain clustering (resolution = 0.6) was then carried out. Cell clusters were annotated on the basis of algorithmically defined marker gene expression for each cluster with *FindAllMarkers* function (method = “Wilcoxon”) along with protein expression data.

### Secondary analysis

Differential gene expression (excluding AbSeq antibodies) was determined using the *FindMarkers* function of the Seurat R package with default parameters, using nonparametric Wilcoxon rank-sum test. Upregulated genes were defined with log_2_-fold change values >1 and downregulated genes by log_2_-fold change values <−1, using a *p*-value cutoff <0.05. Gene ontology over-representation analysis was performed using the *clusterProfiler* (version 4.10.1) package and *enrichGO* function. An adjusted *p*-value cutoff of 0.05 was used. Gene set enrichment analysis, using the *gseGO* function, was performed. Pseudotime and cell trajectory analysis was defined using Monocle2 Plugin on SeqGeq software for HC and PsA Tregs. For monocle analysis, we excluded non-CD4^+^ T-cell AbSeq from the parameters.

### Cell–cell communications

To predict cell–cell interactions and signaling patterns, we applied cell communications CellChat (v2) R package to our single-cell analysis (https://github.com/sqjin/CellChat). CellChat crossreferences a ligand–receptor interaction database (CellChatDB) including extracellular matrix–receptor interactions, cell–cell contact interaction, and secreted signaling. To identify significant cell–cell interactions, differentially expressed ligands and receptors were determined using *identifyOverExpressedGenes* and *identifyOverExpressedInteractions* functions utilizing default parameters. Communications with less than three cells were excluded. Interactions were then associated with a probability value, which was quantified using a mass action–based model. Significant interactions were then determined using a permutation test. To identify cell communication patterns (outgoing and incoming signals), the *ggalluvial* (version 0.12.5) package was used.

### Antibodies and reagents

X-VIVO-20 and X-VIVO-15 media were obtained from Lonza. CD4^+^CD25^+^CD127^lo^ microbeads were from Miltenyi Biotec. All other antibodies (unless otherwise stated) were from BioLegend, eBioscience, or BD Biosciences. CTV was from Invitrogen. AEP Cy5 probe was kindly provided by Prof Matthew Bogyo, Stanford University. AEP inhibitor MV026630 was provided by Prof Colin Watts, Dundee University. Rhapsody cartridge kit, Enhanced cartridge reagent kit, cDNA kit, Human immune response panel, Targeted mRNA & AbSeq kit, AbSeq immune discovery panel CTT LYO, and Human single cell Multiplexing kit were purchased from BD Biosciences and used for single-cell sequencing methods.

### *Ex vivo* culture of lymphocytes

PBMCs were obtained using lymphocyte separation media by density gradient centrifugation. CD4^+^CD25^-^CD127^hi^ cells and CD4^+^CD25^+^CD127^lo^ cells were isolated using a human Treg isolation kit. For AEP activity assays, Tregs were stimulated with phytohemagglutinin (2 μg/ml) along with either anti-PD-1 antibody (20 μg/ml, EH12.2H7) or isotype control (20 μg/ml) and cultured for 3 days. For proliferation culture, PBMCs were labeled with CTV. Briefly, cells were incubated at 37 °C with CTV at 1:1000 dilution in PBS for 20 min. Complete media were added in order to neutralize staining and incubated at 37 °C for 15 min. PBMCs were then centrifuged, washed once with media, and were resuspended in complete media at 1 × 10^6^ cells/ml. About 2 × 10^5^ cells were cultured in 200 ml complete media for 5 days at 37 °C with αCD3 (0.5 μg/ml; BioLegend). A media-only control was used to determine proliferation as a measure of CTV dilution, assessed by flow cytometry.

### AEP activity assay

PBMCs or Tregs were incubated with the AEP cy5 probe LE-28 (1 μM/1 × 10^6^ cells) for 1 h at 37 °C. Cells were then washed twice with PBS. The AEP cy5 probe consists of a quencher that is cleaved by active AEP protein (47). On cleavage, the probe fluoresces which can be identified by flow cytometry. The fluorescence is directly proportional to the amount of AEP activity present within the cells.

### Flow cytometry

PBMCs or expanded Tregs were washed with PBS supplemented with 0.1% bovine serum albumin and 0.01% sodium azide. For cytokine analysis, PBMCs were stimulated for 2 h with cell stimulation cocktail plus protein transport inhibitors (ThermoFisher). Dead cells were excluded from analysis using LIVE DEAD Fixable Blue (ThermoFisher), as per the manufacturer's instructions. Cells were then stained using anti-: CD4 PerCP Cy5.5 (clone: A161A1) or AF700 (clone: SK3), PD-1 BV605 or 650, or APC-cy7 (clone: EH12.2H7), CD48 PeCy7 (clone: BJ40), CD244 BV421 (clone: 2–69), CD16 FITC (clone: B73.1), CD14 PE (clone: M5E2), CD11b APC (clone: ICRF44), CD11c BV605 (clone: 3.9), HLA-DR APC-Cy7 (clone: L243), and TruStain FcX (BioLegend). For intracellular flow cytometry, fixation and permeabilization buffer was utilized (eBioscience). Cells were fixed with the fixation and permeabilization buffer and then stained overnight with FOXP3 PE (clone: 259D, 206D, 236A/E7) or PE/Dazzle (clone: 206D) or AF488 (clone: 150D/E4), IL-10 PE (clone: JES3-19F1), Ki67 BV510 (clone: Ki-67), transforming growth factor-β PerCP Cy5.5 (clone: TW4-9E7), and TruStain FcX. Data were acquired using either a Fortessa or FACS CANTO and analyzed using FlowJo software, version 10.0.6 or 10.9.

### *In vitro* Treg suppression assay

CD4^+^CD25^-^CD127^hi^ cells were isolated using the Miltenyi Biotec Treg isolation kit and utilized in a Treg suppression assay as previously described (48). Purified CD4^+^CD25^-^CD127^hi^ cells (5 × 10^4^) were labeled with CTV and then cultured in 96-well round bottom plates in 200 μl complete media along with Treg suppression inspector beads (Miltenyi Biotec). nTregs were added at indicated ratio either alone or in combination with AEP inhibitor (50 μM). Cells were incubated at 37 °C for 5 days, and proliferation/suppression was monitored by flow cytometry. Proliferation of responder T cells was evaluated by CTV dilution. Percent suppression of CD4 responder T cell was calculated as follows. The positive CD3 control was calculated as 100% proliferation, and then the proliferation of Tregs was determined. This value was then assigned as % proliferation, which was used to calculate % Tregs suppressed.

### Statistical analysis

Flow cytometry data were analyzed using Student’s two-tailed *t* tests. Comparison values of *p* ≤ 0.05 were considered statistically significant. For comparison of cultures within the same patient, a paired test was performed. For comparison between HC and PsA patients, an unpaired Student's *t* test was used. Single-cell data were analyzed in R studio. For violin plots, the Wilcoxon test was used to compare between groups. For box plots generated in R studio, the *FetchData* function was used, using default parameters. Data were then visualized using *ggplot* and Wilcoxon test to compare between groups. Data shown in box plots and violin plots represents gene expression values in the respective groups.

## Data availability

All the data are contained within this article. Single-cell data have been deposited at Gene Expression Omnibus under accession number GSE280319, and the code is deposited in GitHub at https://github.com/teganmct/humanscRNAseq_PSA.

## Supporting information

This article contains supporting information.

## Conflict of interest

The authors declare that they have no conflicts of interest with the contents of this article.
