# Supplementary figures and images for: Deep phenotyping of T regulatory cells in psoriatic arthritis highlights targetable mechanisms of disease
Source: J Biol Chem. 2024 Dec 9;301(1):108059. doi: 10.1016/j.jbc.2024.108059 (PMC11750473; doi:10.1016/j.jbc.2024.108059)

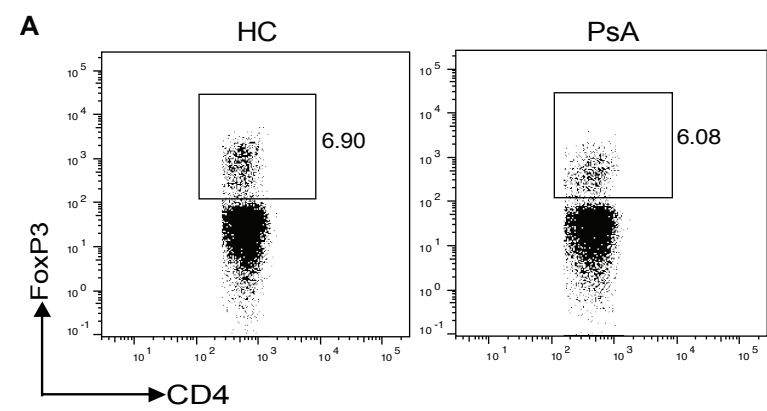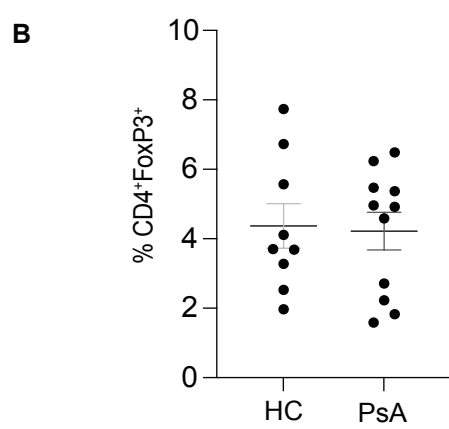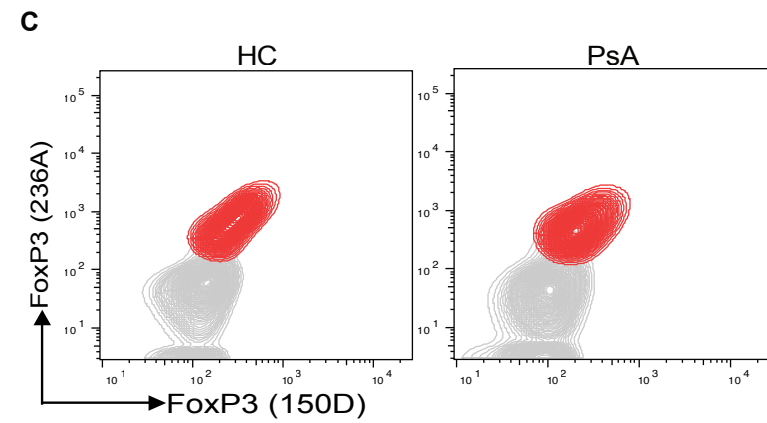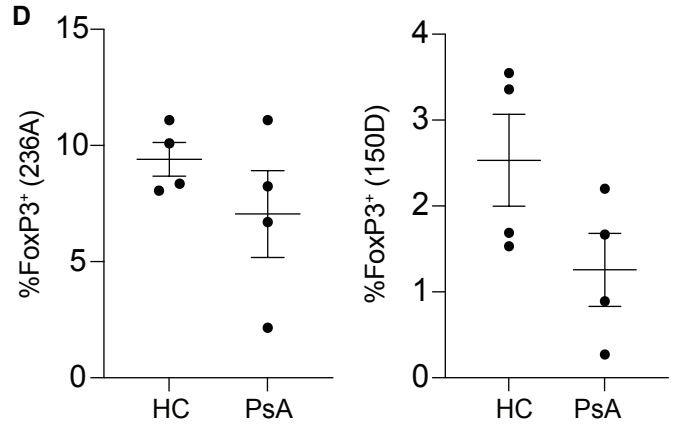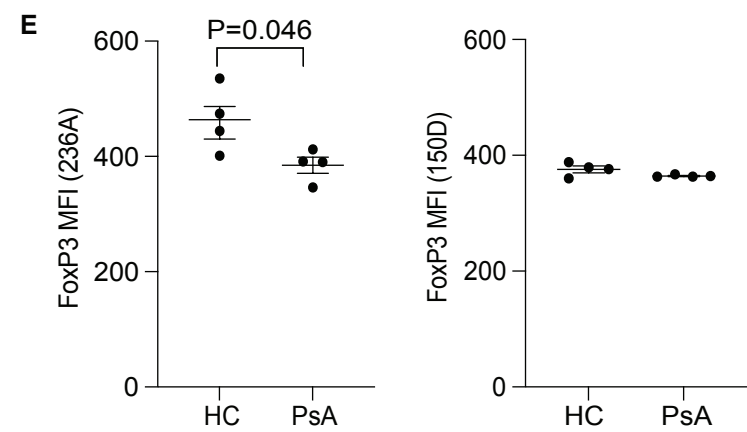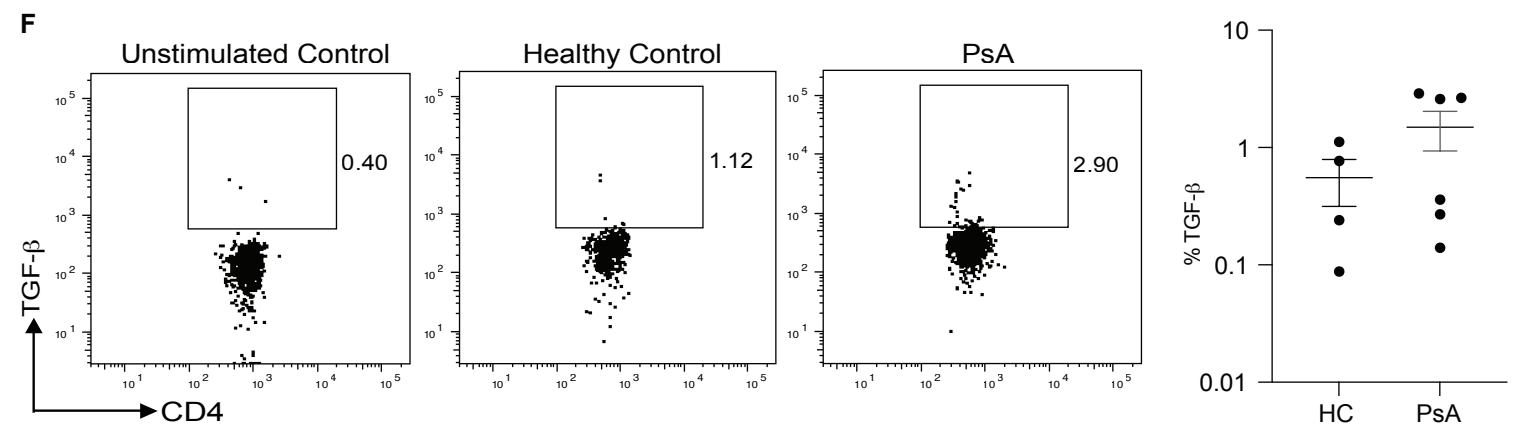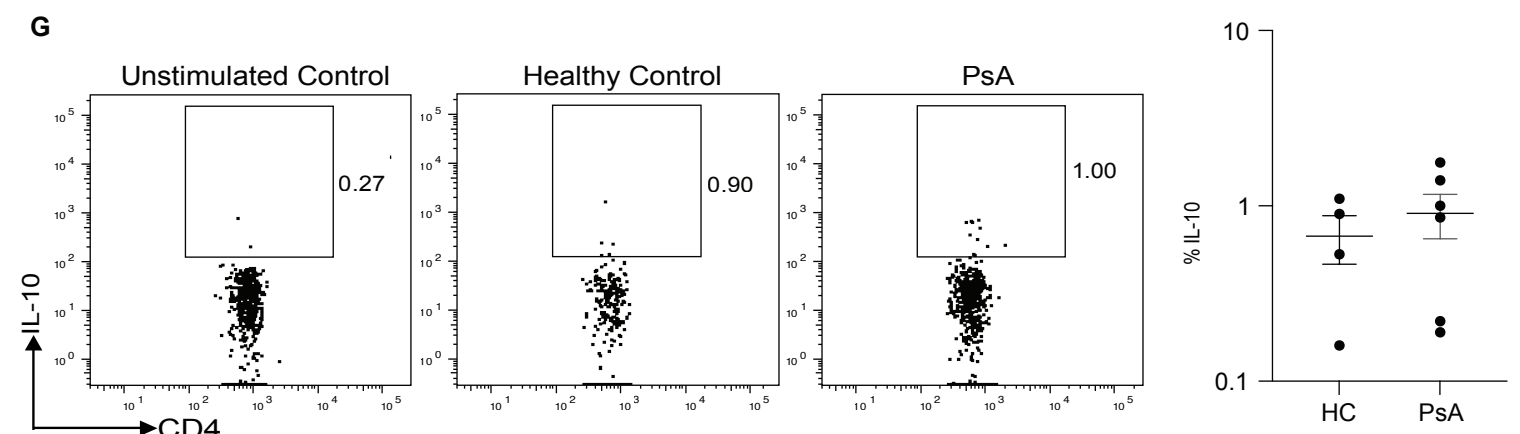

Supplement: Figure S1 [file mmc1.pdf]

**A**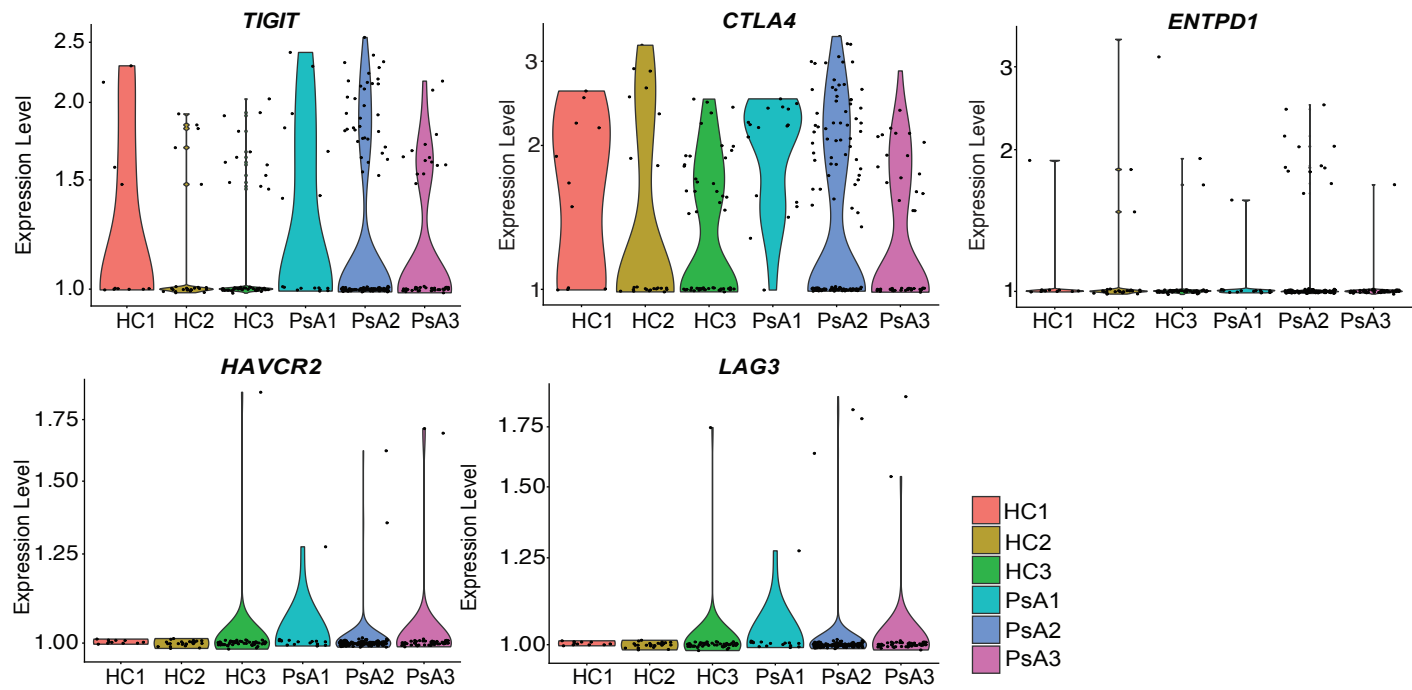**B**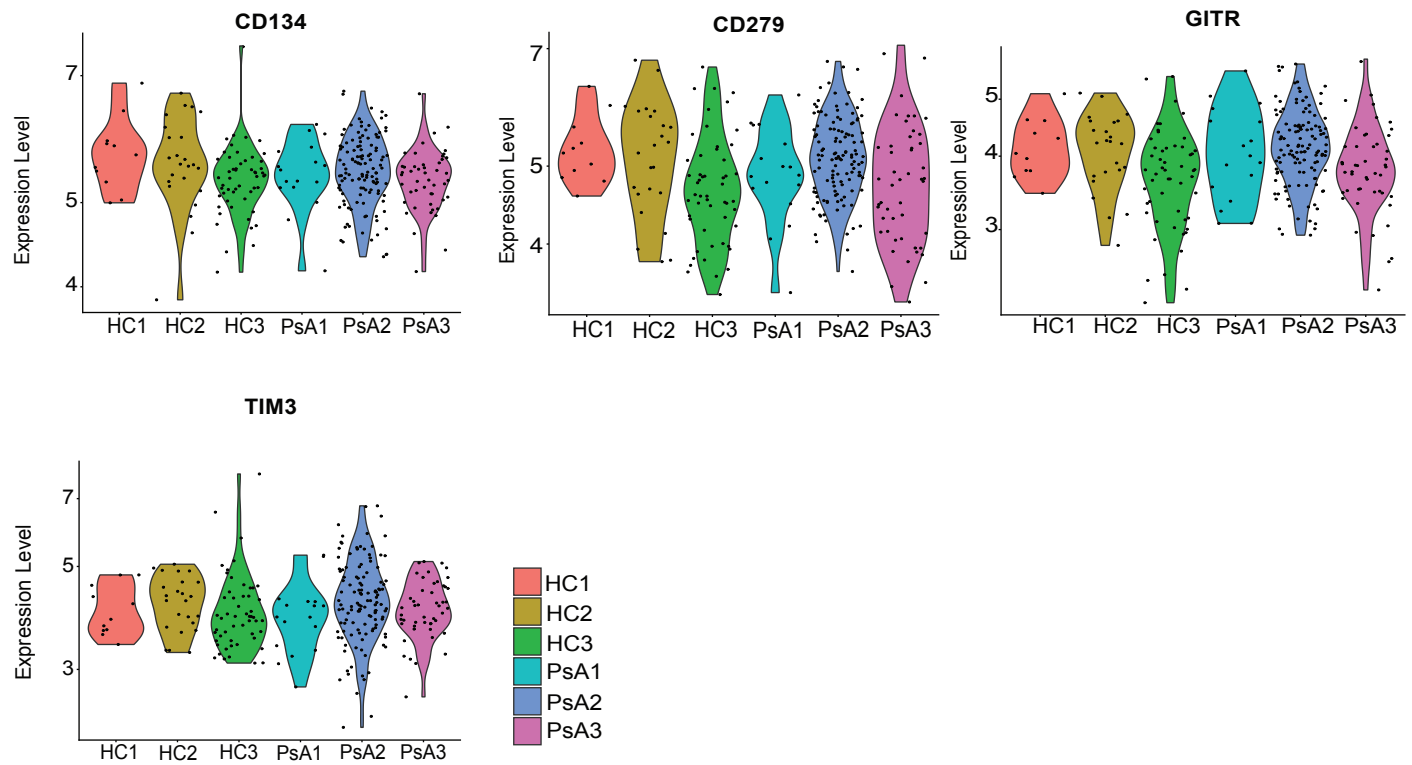**C**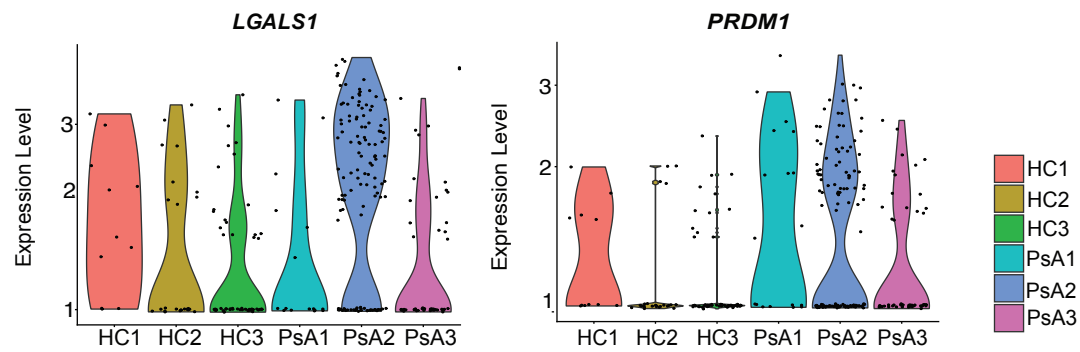

Supplement: Figure S2 [file mmc2.pdf]

Gated on CD4<sup>+</sup>FoxP3<sup>+</sup>

**A**

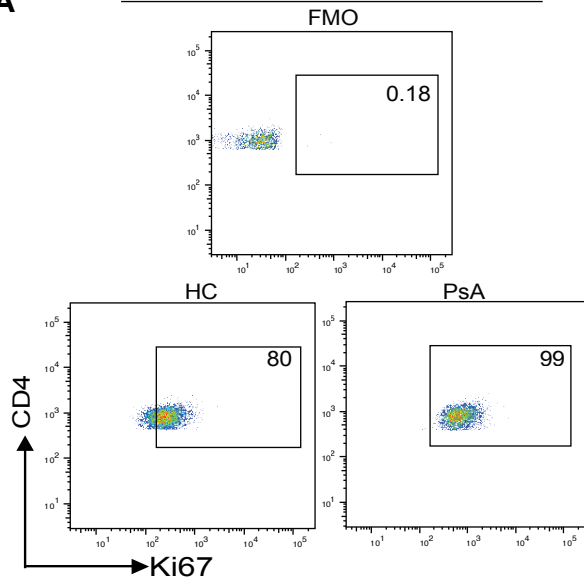

**B**

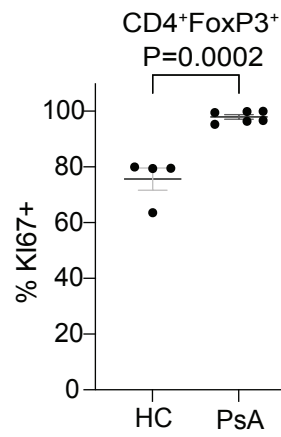

**C**

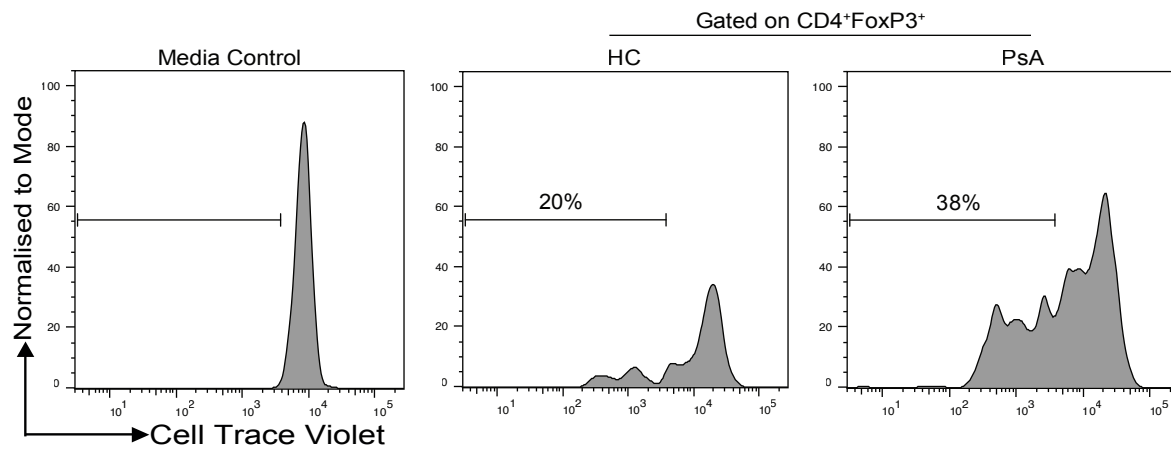

**D**

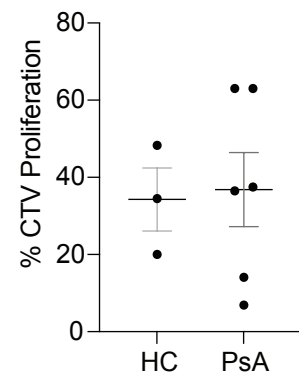

Supplement: Figure S3 [file mmc3.pdf]

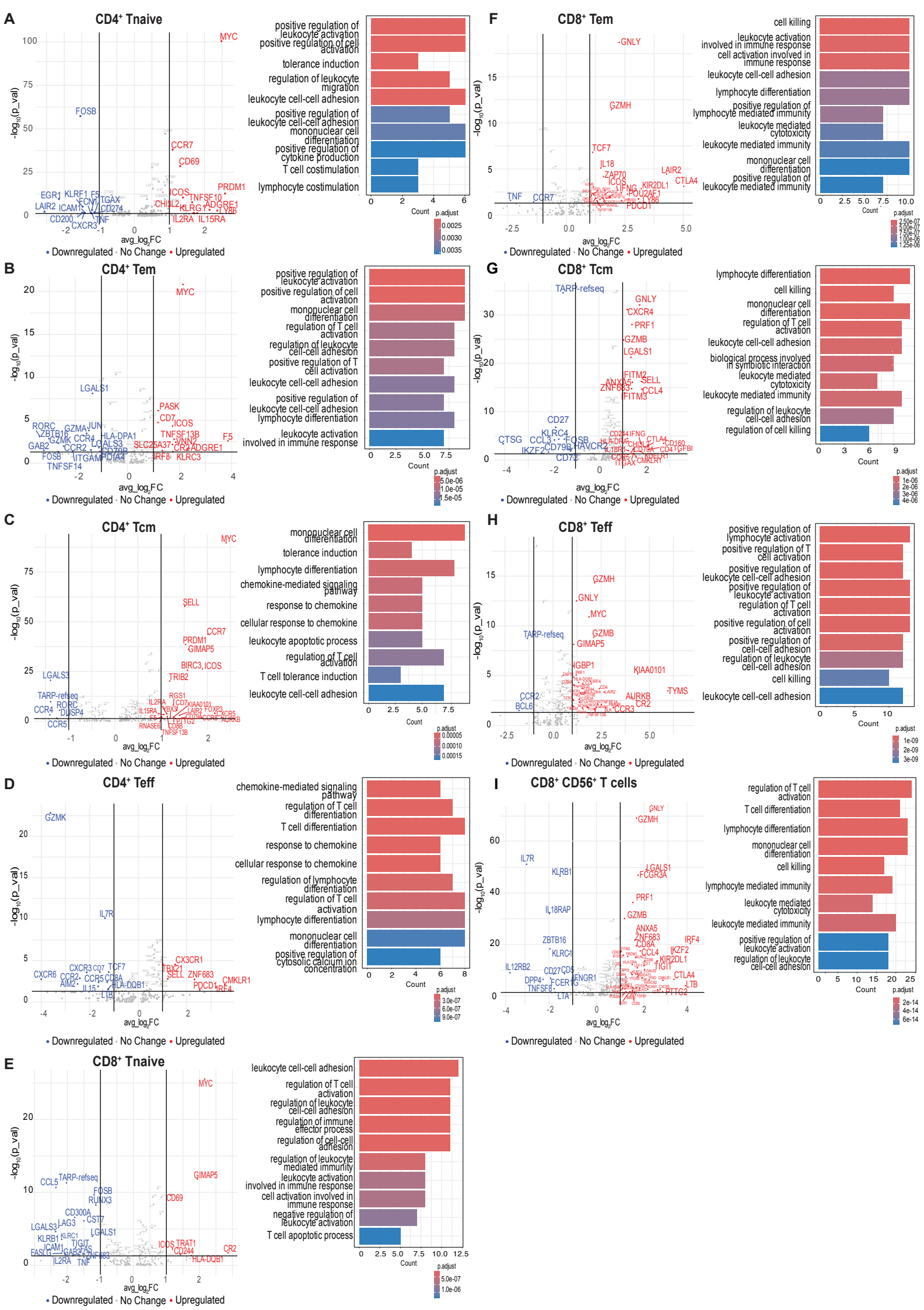

Supplement: Figure S4 [file mmc4.pdf]

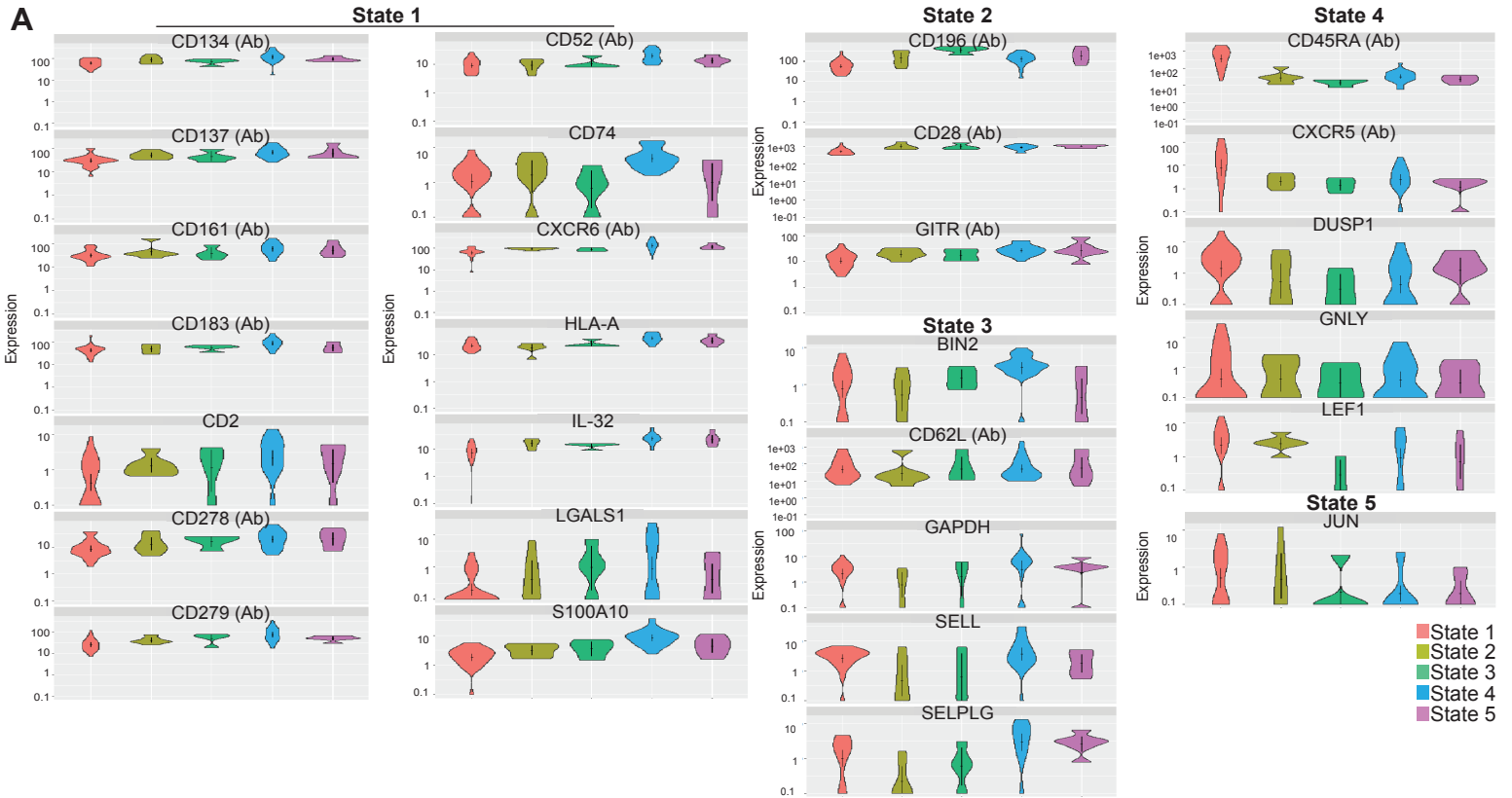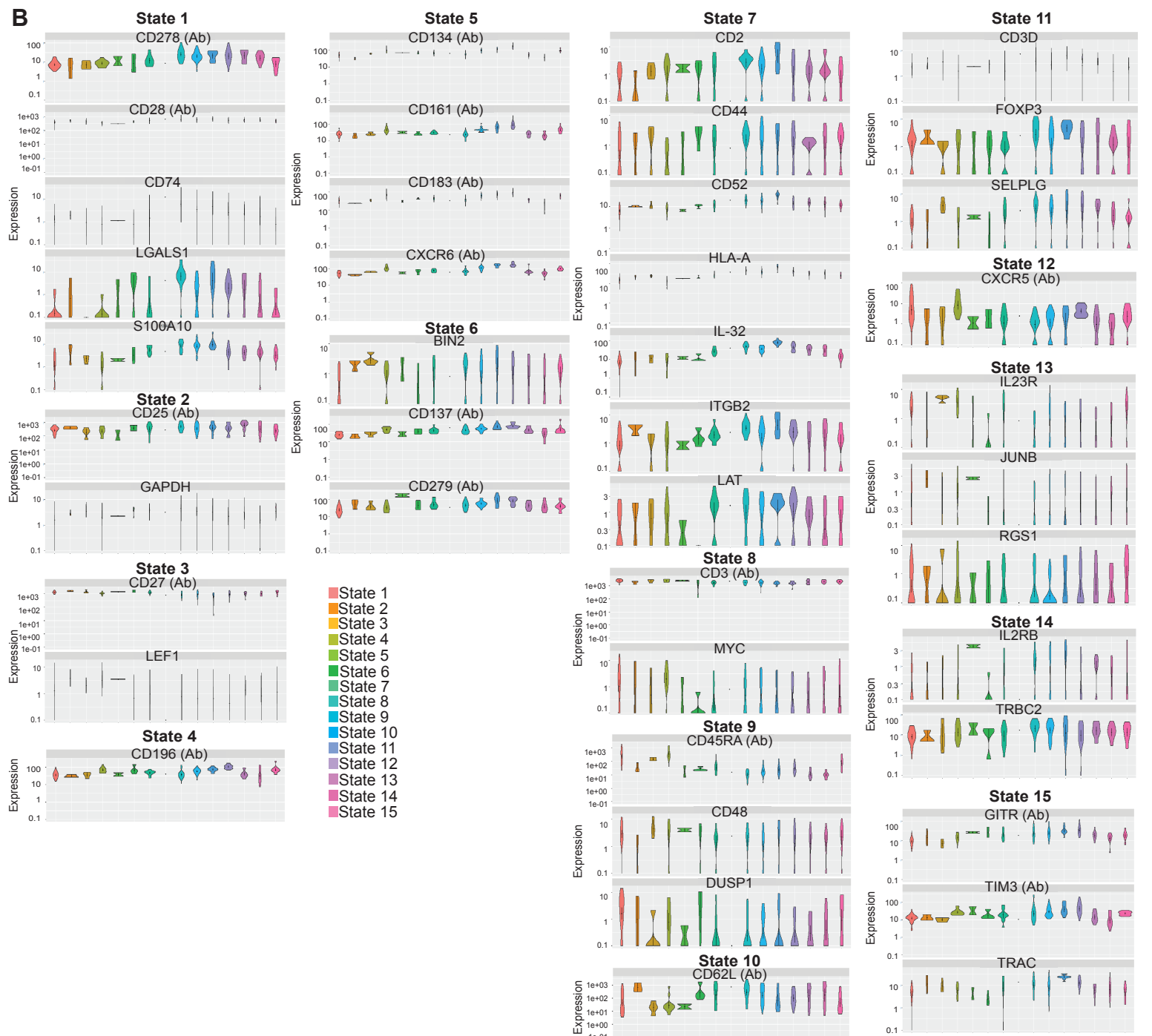

Supplement: Figure S5 [file mmc5.pdf]

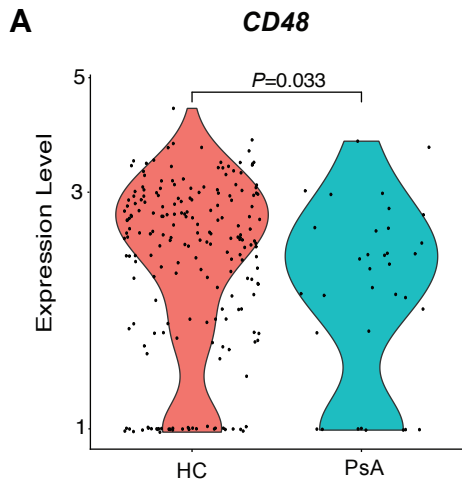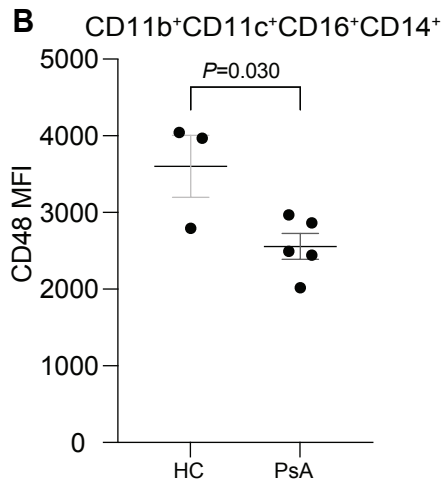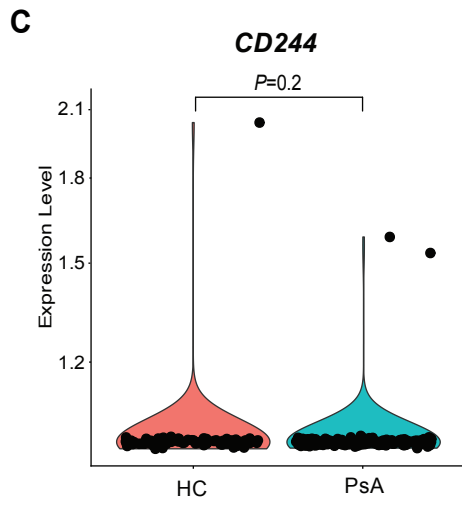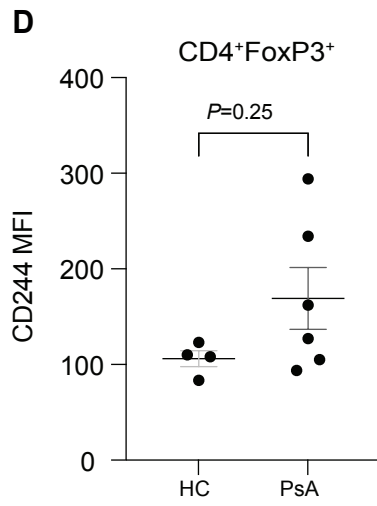

Supplement: Figure S6 [file mmc6.pdf]
