# Supplementary material for: Deep phenotyping of T regulatory cells in psoriatic arthritis highlights targetable mechanisms of disease
Source: J Biol Chem. 2024 Dec 9;301(1):108059. doi: 10.1016/j.jbc.2024.108059 (PMC11750473; doi:10.1016/j.jbc.2024.108059)
Supplement: Supplementary figure legends [file mmc8.docx]

**Deep phenotyping of T regulatory cells in psoriatic arthritis highlights targetable mechanisms of disease.**

**Supplementary Files**

**Supplementary figure 1: Flow cytometry Characterization of Tregs in PsA.**

Human PBMCs were isolated from PsA patients and age-matched healthy controls (HC). PBMCs were stained for CD4 and FoxP3. Representative flow cytometry plots (A) and Treg frequency determined by CD4^+^FoxP3^+^ gated on all CD4^+^ cells (B), n=9 HC and n=11 PsA. FoxP3 exon 2 expression was measured using the 150D/E4 clone against total FoxP3 by flow cytometry, gated on all CD4^+^ cells. Representative flow plot, frequency and mean fluorescence intensity (MFI) of FoxP3 clones in HC and PsA (C-E). PBMCs were stimulated for 2 hrs with cell stimulation cocktail and cytokine analysis was performed by flow cytometry. Representative flow plots of TGF-$\beta$ and frequency (F) and representative flow plots of IL-10 and frequency (G) in n=4 HC and n=5 PsA. Data shown are Mean+SEM and an unpaired Student *t* test was performed to determine statistical significance. Each individual plot signifies an individual donor.

**Supplementary figure 2: Single-cell analysis of Tregs in each individual donor shows similar expression of mRNA and protein expression across samples**

PBMCs from healthy controls (HC) and Psoriatic Arthritis (PsA) were subjected to single cell analysis using 30 AbSeq antibodies and a 399 immune gene panel. Tregs were isolated and separated by donor and co-inhibitory receptor transcript expression is shown (A). Protein expression of coreceptors on Tregs determined by AbSeqs in each individual donor (B). Differentially expressed genes, *LGALS1* and *PRDM1,* from unbiased analysis in each individual donor (C). Data shown are from n=3 samples for HC and PsA.

**Supplementary figure 3: PsA Tregs show enhanced Ki67 expression but possess similar *in vitro* proliferation phenotype to healthy controls**

Human PBMCs were isolated from PsA patients and age-matched healthy controls (HC) and stained for CD4, FoxP3 and the cell cycle marker Ki67. Representative flow plots of Ki67 expression (A) gated on CD4^+^FoxP3^+^ Tregs and the frequency of Ki67^+^ Tregs (B). 2 x 10^5^ PBMCs were labelled with Cell Trace Violet (CTV) and cultured with αCD3 for 5 days. Cells were then stained for CD4 and FoxP3 and analyzed by flow cytometry. Representative flow plots (C) and frequency of proliferation (D) measured by CellTrace Violet dilution, using a media control. Data shown are from n=4 HC and n=5 PsA. Each individual plot signifies an individual donor. Data shown are Mean+SEM and an unpaired Student *t* test was performed to determine statistical significance.

**Supplementary figure 4: Differential gene and Gene Ontology over-representation analysis in CD4^+^ and CD8^+^ cell clusters**

Differential gene expression was calculated for CD4^+^ Naïve T cells (A), CD4^+^ T Effector Memory (Tem)(B), CD4^+^ T Central Memory (Tcm)(C), CD4^+^ Effector T cells (Teff) (D), CD8^+^ Naïve T cells (E), CD8^+^ Tem (F), CD8^+^ Tcm (G), CD8^+^ Teff (H), and CD8^+^ CD56^+^ T cells (I). Volcano analysis of differential gene expression between PsA and HC, and Gene Ontology over-representation analysis in PsA Tregs using adjusted P values are shown. Upregulated (red) and downregulated (blue) genes were defined by log_2_ fold change > 1 or < -1 and a *p* value < 0.05.

**Supplementary figure 5: Differential gene programs in pseudotime derived states in HC and PsA Tregs**

The single-cell data was analyzed using the monocle plugin on SeqGeq software. For each state identified, differential gene expression analysis was performed. In HC Tregs, five different states were observed defined by differential gene expression across Treg states (A). In PsA Tregs, fifteen different gene programs were noted (B), shown as individual gene violin plots with relative gene expression within each state. For monocle analysis, non-CD4^+^ T-cell AbSeq were excluded.

**Supplementary figure 6: CD48 and CD244 transcript and protein expression on Tregs**

CD11b^+^CD11c^+^CD16^+^CD14^+^ OCPs were isolated from single-cell RNAseq data and analyzed for *CD48* transcript expression (A). PBMCs were isolated from PsA patient blood and age-matched healthy controls (HC) and analyzed by flow cytometry. Mean fluorescence intensity (MFI) for CD48 protein on OCPs defined by CD11b^+^CD11c^+^CD16^+^CD14^+^ was calculated using geometric mean (B). Tregs were isolated from single-cell RNAseq data and analyzed for *CD244* transcript expression (C). Protein expression of CD244 on Tregs (D), measured by flow cytometry. Data shown are Mean+SEM. Statistical analysis for violin plots were done using the Wilcoxon test and for flow cytometry an unpaired Student *t* test was used. Each individual plot signifies an individual donor.
